# Socioeconomic Determinants of COVID-19 Incidence and Mortality in Florida

**DOI:** 10.7759/cureus.22491

**Published:** 2022-02-22

**Authors:** Sean Backer, Aida Rezene, Payal Kahar, Deepesh Khanna

**Affiliations:** 1 Osteopathic Medicine, Nova Southeastern University Dr. Kiran C. Patel College of Osteopathic Medicine, Clearwater, USA; 2 Osteopathic Medicine, Nova Southeastern University Dr. Kiran C. Patel College Of Osteopathic Medicine, Clearwater, USA; 3 Epidemiology and Public Health, Florida Gulf Coast University, Fort Myers, USA; 4 Epidemiology and Public Health, Nova Southeastern University Dr. Kiran C. Patel College Of Osteopathic Medicine, Clearwater, USA

**Keywords:** centers for disease control(cdc), cdc coronavirus, covid-19 symptoms, covid-19 vaccine, mortality index, general infectious diseases, socio-economic factors, corona virus, corona virus disease 2019, covid 19

## Abstract

A wide variety of social determinants of health have been associated with various risks and impacts on quality of life. Specifically, poverty, lack of insurance coverage, large household sizes, and social vulnerability are all factors implicated in incidence and mortality rates of infectious disease. However, no studies have examined the relationship of these factors to the COVID-19 pandemic on a state-wide level in Florida. Thereby, the objective of this study is to examine the relationship between average household size, poverty, uninsured populations, social vulnerability index (SVI), and rates of COVID-19 cases and deaths in Florida counties.

The objective was accomplished by analyzing the cumulative case and death reports from state and local health departments in Florida. The data was compiled into a single dataset by the CDC COVID-19 Task Force. Using US Census Bureau data, all Florida counties were classified into tertiles of the separate categories of poverty rate, average household size, uninsured rates, and SVI (Social Vulnerability Index). The poverty level was classified as low (0-12.3%), moderate (12.3-17.3%), and high (>17.3% below the federal poverty line). The uninsured population proportion was classified as low (0-7.1%), moderate (7.1-11.4%), and high (>11.4% uninsured residents). Average county household size was classified as low (0-2.4), moderate (2.4-2.6), and high (>2.6). The Centers for Disease Control and Prevention (CDC)/Agency for Toxic Substances and Disease Registry (ATSDR) Social Vulnerability Index (SVI) used US census data on 15 social determinants of vulnerability to evaluate and assist disadvantaged communities. SVI tertiles were low (0-0.333), moderate (0.334-0.666), and high (0.667-1) on a range of 0-1, with higher numbers signifying communities with many factors of social vulnerability. The mean cumulative cases and deaths per 100,000 inhabitants were calculated in each tertile for each category.

Analysis of the data revealed that case and mortality rates due to COVID-19 in the high poverty counties were markedly higher in Florida than the national average. In contrast, moderate and low poverty rates were below average. Similarly, counties with a high SVI had case and mortality rates greatly above state and national averages. Counties with a high proportion of uninsured displayed the highest case rates. However, mortality rates were the highest in counties with a low proportion of uninsured individuals. No clear correlation was observed between COVID-19 rates and household size.

It was concluded that compiled CDC and US census data suggests a significant correlation between poverty, social vulnerability, lack of insurance coverage, and increased incidence and mortality from COVID-19. Future research should statistically analyze the correlations and examine the individual factors of SVI as potential COVID-19 predictors.

## Introduction

Believed to originate from initial zoonotic transmission to a human in Wuhan, China, in late 2019, the SARS-CoV-2 virus has now produced a pandemic of global significance [1,2]. COVID-19 is caused by SARS-CoV-2, which now undergoes widespread community transmission via aerosol, droplet, contact, and fomite transmission [3,4]. The spread and impact of the pandemic are particularly evident in the United States. Within four months of arrival in the United States, the virus had spread to all 50 states and 90% of all counties [5]. As of January 13, 2022, the CDC reports approximately 63,397,935 total cases and 842,873 COVID-19-related deaths in the United States [6]. According to the data in Dec 2021, Florida accounts for 4,933,000 cases and 63,000 deaths alone, making it third among all US states in the total number of cases and deaths. Florida has the third-highest case rate per 100,000 inhabitants (22,970) among all US states [6]. With such a significant transmission, Florida is an ideal cohort to examine how various demographic factors influence rates of COVID-19 cases and deaths. Socioeconomic status may have a significant impact on COVID-19 impact on various communities. For instance, one study of different zip-codes in Miami-Dade County found a significant correlation between median household incomes, self-reported financial stress, and rates of COVID-19 infections [7]. Even in models adjusted for population density and household size, decreases in median household income were correlated to increased infection rates [7].

Generally, two issues are addressed when explaining the observed correlation between socioeconomic status and COVID-19 impact. Firstly, it is believed that COVID-19 disproportionately affects lower-income communities due to the inability to distance correctly socially [8,9]. The privilege of social distancing is often compromised in lower-income populations since maintaining a sufficient income by working remotely is rarely a feasible option [9]. Furthermore, lower-income populations are more likely to work in public-facing service occupations [8]. In addition to the increased risk of exposure through professional settings, household transmission in lower-income communities may also contribute.

Interestingly, an observational cohort study in the United Kingdom found that household size was not significantly correlated to incidences of positive COVID-19 test results during times without lockdown [7,10]. However, during times of mandatory lockdown, the UK study did find a significant correlation between larger households and increased incidence of COVID-19 cases [10]. This may further emphasize the privilege of social distancing. Employed members of larger, low-income households may still be obligated to attend physical work during the lockdown. Upon returning to a larger household, the transmission rate may subsequently be amplified further within that community.

Additionally, a lack of access to healthcare may also contribute to increased severity and mortality from COVID- 19, particularly in a US healthcare system with a large uninsured population. A study conducted in New York examined uninsured individuals between 18 and 64 years old and found that neighborhoods with a high proportion of uninsured individuals had significantly increased COVID-19 positive rates. In fact, the uninsured proportion was the most or second most important predictive variable in predicting positivity rate in univariate analysis [11]. In addition to uninsured status serving as an essential factor in COVID-19 outcomes, it is also a significant public health variable due to the large population it involves. In a recent issue brief (February 11, 2021) of the National Health Interview Survey (by the Assistant Secretary for Planning and Evaluation), it was reported that Florida has among the largest uninsured populations in the United States with 2,860,759 uninsured, nonelderly individuals, making up 16.8% of the total nonelderly population [12]. Although the data is descriptive, it is essential to note the exclusion of the elderly population, as they are a high-risk population in the COVID-19 pandemic.

Overall, a wide range of factors may predispose specific disadvantaged communities to higher rates of COVID-19 infection, severity, and mortality. These numerous components can be analyzed by examining the Social Vulnerability Index. The CDC/ATSDR (agency for toxic substances and disease registry) Social Vulnerability Index (SVI) was developed by the Geospatial Research, Analysis, and Services Program (GRASP) to assess how to meet the needs of vulnerable populations during natural disasters or public health emergencies (such as infectious disease outbreaks) [13]. The SVI is composed of US census data on 15 social factors divided into socioeconomic status, household composition and disability, minority status and language, housing type, and transportation. A 2020 statistical analysis of CDC/ATSDR SVI concerning COVID-19 cases and deaths, on a national level (reported by New York Times as of April 2020), demonstrated a 1.63-fold increased risk of COVID-19 diagnosis and 1.73-fold increased mortality rate in the high SVI tertile relative to the low vulnerability tertile [14]. In particular, the study found that the SVI categories of socioeconomic status and, housing type & transportation were associated with increased case rates and mortality in urban areas.

In contrast, minority status and language was the predominant factor of COVID-19 aggravation in rural counties [14]. Another study found a significant correlation between overall SVI and the minority status & language subcategory to increased COVID-19 case rates [15]. Social Vulnerability Index is an essential composite of factors that may determine COVID-19 outcomes on the county level.

As evidence, there is a high volume of research on the relationship between socioeconomic status and its various components to COVID-19. Generally, it is proposed that inability to socially distance, lack of healthcare access, and increased social vulnerability in low-income communities mediate higher transmission and mortality in low-income communities. A correlation between socioeconomic status and COVID-19 cases in Miami-Dade County has been detailed, yet there are no studies on a state-wide level in Florida [7]. Additionally, the specific data of household size, uninsured rate, and social vulnerability about both cases and deaths in Florida has not been presented comprehensively. This study will provide a comprehensive analysis of CDC data on the number of COVID-19 cases and deaths in Florida counties to the prevalence of poverty, uninsured individuals, social vulnerability index, and household size.

## Materials and methods

Study population

The dataset on COVID-19 cases and deaths in Florida was based on daily reports from state and local health departments, compiled by the Centers for Disease Control and Prevention (CDC), between January 23, 2020, and January 13, 2022. Health departments in all 50 US states and territories have been mandated to report all COVID-19 cases and casualties to the CDC. Despite the mandatory reporting, the CDC suggests potential errors with accuracy due to infected individuals delaying or not seeking medical care and variations in procedures for reporting cases and deaths. COVID-19 cases are reported and dated based on the positive test date, specimen sampling, or clinical diagnosis. The data on deaths are reported and dated based on the date of death provided in official death certificates from COVID-19 associated casualties. The study population includes all individuals with a positive COVID-19 diagnosis or death reported in Florida [6].

Measures

Demographic data on poverty level, household size, social vulnerability index, and health insurance status was compiled by the Centers for Disease Control and Prevention (CDC) by American Community Survey data. The CDC then used the compiled survey data to classify all Florida counties into distinct tertiles, denoted as low, moderate, and high, in each of the aforementioned demographic categories. For instance, the poverty level of Florida counties was classified as the tertiles low (0-12.3% of households in poverty), moderate (12.3-17.3%), and high (>17.3% of households below the poverty line), based on the federal poverty income level for a given household. Similarly, by survey data on health insurance status (proportion uninsured), household size, and CDC/ATSDR Social Vulnerability Index, counties were also categorized into tertiles of the lowest, intermediate, and highest proportions among their residents. The tertiles for uninsured proportion per county were low (0-7.1%), moderate (7.1-11.4%), and high (>11.4% uninsured residents). The tertiles for average county household size were low (0-2.4), moderate (2.4-2.6), and high (>2.6). The tertiles for the social vulnerability index were low (0-0.333), moderate (0.334-0.666), and high (0.667-1) on a range of 0-1, with higher numbers signifying communities with many factors of social vulnerability. Any rates of demographic data used by the CDC were calculated using the US Census Bureau Population Estimates Program [6].

**Figure 1:**
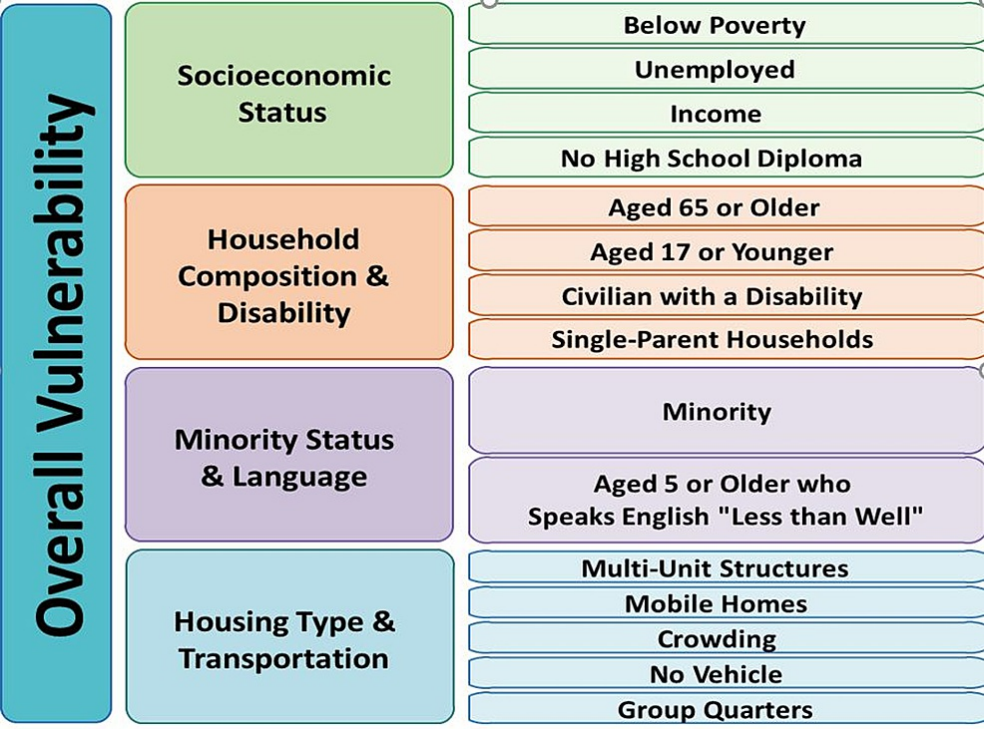
A summary of the categories of and individual social factors that compose the CDC/ATSDR Social Vulnerability Index. [13]

The CDC/ATSDR, Social Vulnerability Index, is composed by the Geospatial Research, Analysis, and Services Program (GRASP) and utilizes US census data on 15 social determinants of vulnerability to examine disadvantaged communities and evaluate an appropriate response in public health emergencies and disasters. These 15 social determinants are: below poverty, income, no high school diploma, unemployed, aged 65 or older, aged 17 or younger, civilian with a disability, single-parent households, minority status, aged five or older and speaks English “less than well”, multi-unit residency, mobile home, crowding, and no vehicle. Figure 1 is obtained from the Agency of Toxic Substances and Disease Registry and illustrates the classification of social factors that compose CDC/ATSDR SVI13.

Statistical analysis

A statistical analysis of significant difference, such as a 2-sample t-test, could not be performed on the data since variance and standard deviations could not be performed. Although the CDC Data Tracker provided access to county-level data for all US counties (including all Florida counties), there was no information on which counties were included in the CDC-determined tertiles for each independent variable (poverty, household size, uninsured rate, SVI). Thus, it was not possible to correctly assign standard deviations within the sample group tertiles.

Instead of analyzing significant differences, the differences between the highest dependent variable results and the FL and national averages were calculated and presented as proportional (in %) differences. This was mainly done to show data findings in a more presentable manner. Thereby, it should be noted that although some results showed large increases compared to FL/national averages, these results may or may not be statistically significant, as the variance is unaccounted for.

## Results

Results were obtained using survey data reported by state and local health departments and compiled by the CDC on COVID-19 cases and deaths reported in Florida, from January 23, 2020, to January 13, 2022. The COVID-19 case and death distributions based on each demographic are depicted in Figures 2-9 [6].

**Figure 2:**
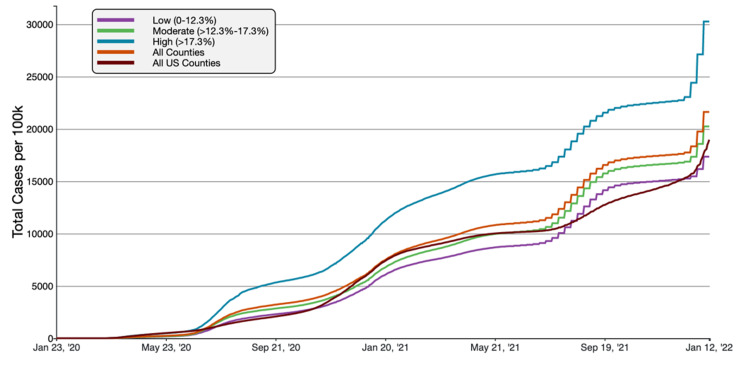
COVID-19 Cumulative Case Rate per 100,000 Population in Florida, by Percentage of County Population in Poverty A graphical representation of COVID-19 case rate per 100 000 inhabitants in tertiles of Florida counties based on poverty in comparison to all FL and US counties. This figure was obtained from the CDC website on Jan 13th, 2022 [6].

**Figure 3:**
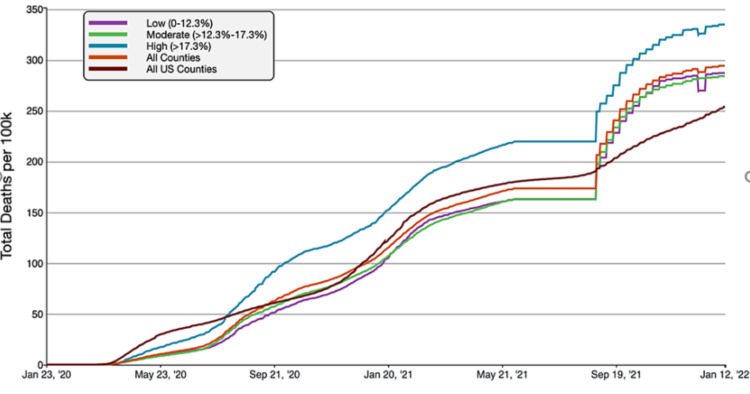
COVID-19 Cumulative Death Rate per 100,000 Population in Florida, by Percentage of County Population in Poverty A graphical representation of COVID-19 death rate per 100,000 inhabitants in tertiles of Florida counties based on poverty in comparison to all FL and US counties. This figure was obtained from the CDC website on Jan 13th, 2022 [6].

**Figure 4:**
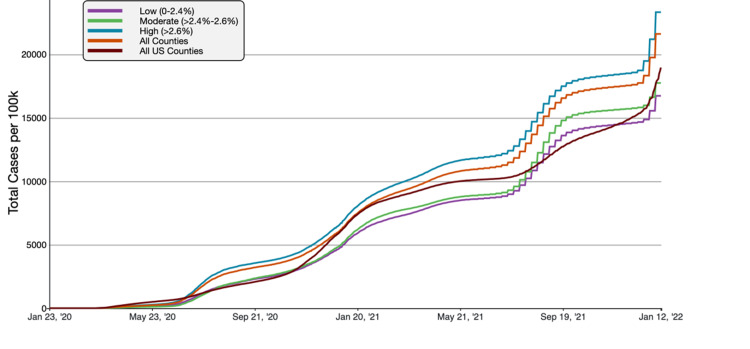
COVID-19 Cumulative Case Rate per 100,000 Population in Florida, by County Average Household Size A graphical representation of COVID-19 case rate per 100,000 inhabitants in tertiles of Florida counties based on average household size in comparison to all FL and US counties. This figure was obtained from the CDC website on Jan 13th, 2022 [6].

**Figure 5:**
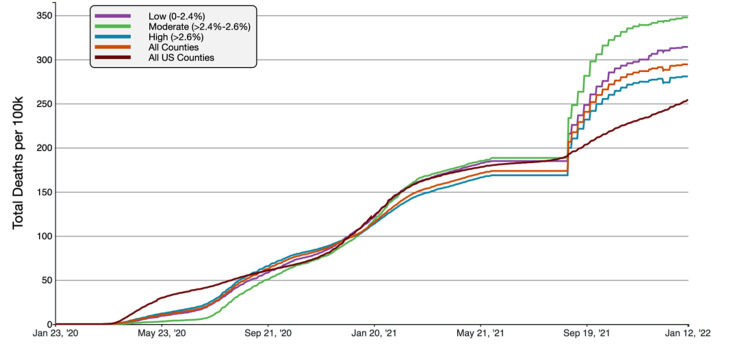
COVID-19 Cumulative Death Rate per 100,000 Population in Florida, by County Average Household Size A graphical representation of COVID-19 death rate per 100,000 inhabitants in tertiles of Florida counties based on average household size in comparison to all FL and US counties. This figure was obtained from the CDC website on Jan 13th, 2022 [6].

**Figure 6:**
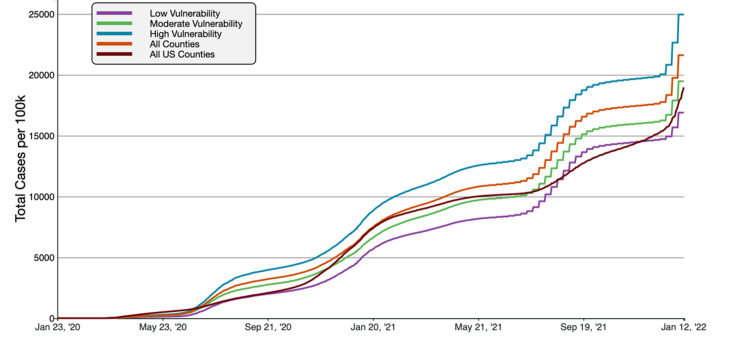
COVID-19 Cumulative Case Rate per 100,000 in Florida, by county Social Vulnerability Index Value A graphical representation of COVID-19 case rate per 100,000 inhabitants in tertiles of Florida counties based on CDC/ASTDR’s social vulnerability index in comparison to all FL and US counties. This figure was obtained from the CDC website on Jan 13th, 2022 [6].

**Figure 7:**
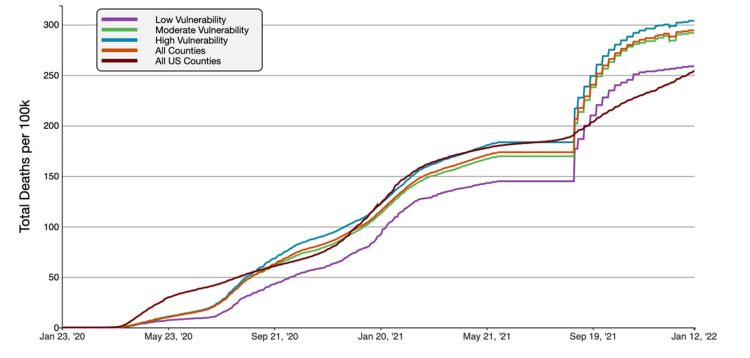
COVID-19 Cumulative Death Rate per 100,000 in Florida, by county social Vulnerability Index Value A graphical representation of COVID-19 death rate per 100,000 inhabitants in tertiles of Florida counties based on CDC/ASTDR’s social vulnerability index in comparison to all FL and US counties. This figure was obtained from the CDC website on Jan 13th, 2022 [6].

**Figure 8:**
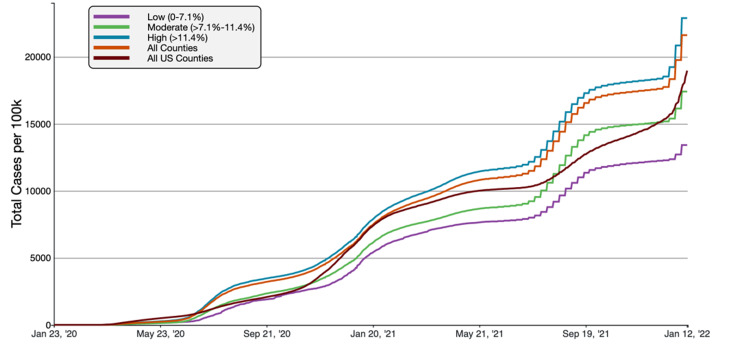
COVID-19 Cumulative Case Rate per 100,000 Population in Florida, by Percentage of County Population Uninsured A graphical representation of COVID-19 case rate per 100,000 inhabitants in tertiles of Florida counties based on uninsured populations in comparison to all FL and US counties. This figure was obtained from the CDC website on Jan 13th, 2022 [6].

**Figure 9:**
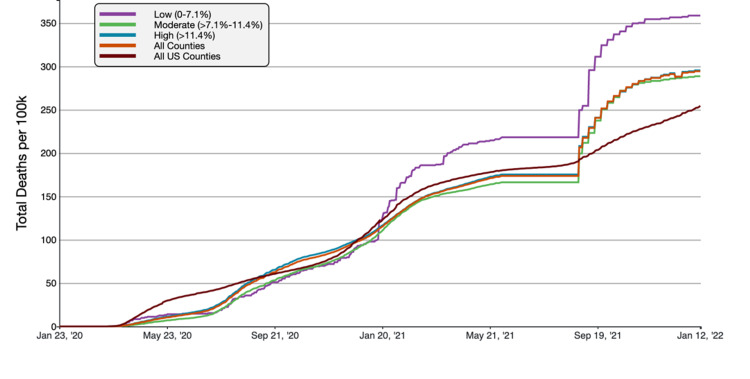
COVID-19 Cumulative Death Rate per 100,000 Population in Florida, by Percentage of County Population Uninsured A graphical representation of COVID-19 death rate per 100,000 inhabitants in tertiles of Florida counties based on uninsured populations in comparison to all FL and US counties. This figure was obtained from the CDC website on Jan 13th, 2022 [6].

Florida counties with the highest percentage of poverty (>17.3% below the federal poverty line) had the highest rate of cumulative COVID-19 cases and deaths per 100,000 people. As of January 13th, 2022, the high-poverty average cumulative cases were 30,259.11 cases/100,000, compared to 21,613.49 cases/100,000 inhabitants and 18,965.72 cases/100,000 in all FL and US counties respectively. Similarly, the mortality rate was 335.00 deaths/100,000 in high-poverty counties, compared to 294.32 deaths/100,000 inhabitants in all Florida counties and 254.48 deaths/100,000 in all US counties. Overall, high-poverty FL counties had 40.00% and 59.54% greater case rates than FL and national average, respectively. The mortality rate was 13.82% and 31.64% higher than Florida and the national average.

COVID-19 cumulative case rates were the highest in the large household size group, and the cumulative death rate was the highest in moderate household size counties as of November 21, 2021. Large household counties had an average of 23,328.24 cases/100,000, compared to 21,613.49 cases/100,000 and 18,965.72 cases/100,000 in all Florida counties and US counties respectively. Concerning the Florida average, a 7.93% increase and 23.00% higher than the national average. For cumulative death rates, moderate size household counties had 347.57 deaths/100,000 inhabitants, compared to 294.32 deaths/100,000 and 254.48 deaths/100,000 inhabitants in all Florida counties and US counties. The moderate household counties had an 18.09% increased mortality rate than the Florida average and a 36.58% higher value than the national average.

Using the CDC/ASTDRs SVI to measure social vulnerability to COVID-19, counties with a high SVI (0.667-1) were found to have the highest cumulative cases and deaths rates as of January 13, 2022. The high SVI group had an average cumulative case rate of 24,949.88 cases/100,000, compared to 21,613.49 cases/100,000 and 18,965.72 cases/100,000 inhabitants in all Florida counties and US counties, respectively. The cumulative death rate was 303.76 deaths/100,000, to all Florida counties at 294.32 deaths/100,000 and all US counties at 254.48 deaths/100,000. The high SVI (vulnerable) Florida counties had case rates 15.44% higher than the FL average, and death rates were 3.21% higher. To the national average, case rate and mortality were increased by 31.55% and 19.36%, respectively.

Cumulative COVID-19 cases rates were the highest in high-uninsured counties (>11.4% uninsured), whereas cumulative death rates were highest in counties with a low uninsured population (<7.1% uninsured). The high-uninsured counties had a cumulative case rate of 22.885.04 cases/100,000, compared to 21,613.49 in Florida overall and 18,965.72 nationally. The high-uninsured counties had a 5.88% higher case rate than average in Florida and 20.67% higher than the national average. The low-uninsured counties had the highest mortality at 358.64 deaths/100,000, compared to 294.32 in all Florida counties and 254.48 nationally. The mortality rate in low-uninsured counties was 21.85% higher than the FL average and 40.93% higher than the national average.

## Discussion

The COVID-19 pandemic has placed an unprecedented burden on both a nationwide and global scale, revealing how the rates of infection and mortality have been exacerbated by the variables reviewed in this comprehensive analysis. Florida specifically appears to have been among the most negatively impacted states throughout this pandemic, at one point even accounting for 20% of new infections nationally [16].

As previously mentioned, a relationship was observed between the rate of COVID-19 cases and deaths in low-income populations in Florida counties. High poverty counties exhibited higher rates of cases and deaths when compared to the state and national averages. According to one US-county cross-sectional analysis that looked at how community, household, and individual influences impact COVID-19, Florida appeared to be one of the most impacted states to higher poverty rates, which supports our findings and provides reason to believe that the state has been disproportionately affected [17]. While reasons for this may vary, previous literature suggests that the combination of overcrowded homes and unsanitary living conditions in low-income houses make social distancing difficult and increases the risks of other respiratory infections. Poorer individuals are often forced to participate in risky health behaviors that include occupations with high levels of public contact and seeking medical care at later stages of diseases to avoid unnecessary costs [18,19]. Not being able to work from home may increase your exposure to the disease while seeking care later, likely resulting in worse outcomes from COVID-19. Moreover, it seems impoverished populations often fear being discriminated against or embarrassed in healthcare facilities due to previous experience with bias in these settings, deterring them from seeking medical care [19].

In addition, according to Wolfson and Leung, 44% of low-income adults were food insecure, supporting the clear disproportionate effect this pandemic has on low-income households, who already are having a difficult time meeting their basic needs [20]. Living in poverty can contribute to poor hygiene and prevent access to clean water [21]. Proper hygiene and sanitation are necessary for controlling and combating SARS-CoV-2. Yet, many low-income countries within South America, Asia, and Africa cannot afford social distance, hand washes properly, or properly dispose of wastes, making it more challenging to mitigate the spread [22]. Furthermore, the combination of effects resulting from financial insecurity is thought to impact one’s mental health and increase stress levels negatively. As a result of the inverse relationship between a functioning immune system and stress, individuals experiencing financial distress are often more susceptible to infection and less capable of mounting an adequate immune response [19].

We looked to see if the same trends in the United States were found elsewhere in the world. In a longitudinal study that collected household survey data from four low-income countries: Nigeria, Uganda, Ethiopia, and Malawi, 77% of the population reported some variation of lost income since the pandemic began. Researchers also discovered increased food insecurity and the inability to meet basic needs such as soap or medicine, making the fight against COVID-19 more difficult [23]. Economically disadvantaged populations are especially vulnerable to the effects of COVID-19. Policymakers should look at ways to ensure the basic needs of individuals are met and how to address these apparent inequalities.

Contrary to what was predicted, our results revealed that the average household size did not appear to have a consistent correlation on rates of cases and deaths. Large household sizes displayed the highest cumulative case rates at 23.00% higher than the national average. While we expected similar trends for death rates, it seemed moderate household sizes had the greatest mortality rate, at a staggering 36.58% higher than the national average. However, these disparities were lower within Florida, as large households only had 7.93% increased case rates, and moderate-sized households had an 18.09% elevation in mortality compared to Florida averages. These results are inconsistent with other literature that suggests a strong positive correlation between mortality rates from COVID-19 and overcrowding on a county level in the United States which implies larger households would experience more deaths from COVID-19 [24,25]. Reasons for this may include but are not limited to increased susceptibility due to lack of social distancing, and thus higher rates of transmission increased contact with infected individuals [24]. A separate cross-sectional analysis conducted in the US found that average household size to be statistically significant (p<0.0001) and the strongest independent predictor of COVID-19 cases and deaths in comparison to other variables studied such as educational attainment, employment, use of public transport, and citizenship status, further reinforcing the impact household size may have [25]. Likewise, research in England and Wales revealed a positive correlation between average household size and infection rates and mortality. Like our study, it was found that larger average household sizes demonstrated higher cases of infection per 100,000 people, but interestingly determined that household size did not have as strong of an association with mortality rates [26,27]. Our findings in conjunction with previous literature lead us to believe that while average household size appears to impact COVID-19, there is no clear correlation. Researchers should focus on ways to alleviate the higher burden on larger households and explore how other factors such as household composition (i.e., more children, elderly individuals) may be responsible for our unlikely findings.

Due to COVID-19, socially disadvantaged communities have been disproportionately affected in their morbidity and mortality rates due to various inequalities in their lifestyles [15]. Our comprehensive analysis revealed a significant relationship between the rate of COVID-19 cases and deaths and Florida counties with a higher SVI (0.667-1). Specifically, the high SVI FL counties case and death rates were found to be 31.55% and 19.36% higher than the Florida average, respectively, suggesting the 15 variables that make up the SVI play a crucial role in COVID-19 outcomes. An idea further supported by a Louisiana study that looked at the four themes of SVI (Figure 1) to the number of COVID-19 cases in each census tract. They found an overall positive correlation between cumulative rates of COVID-19 and the four SVI categories [21]. This idea reflects and reinforces a long history of adverse health outcomes due to social determinants of health within the United States. Pandemics have been disproportionately affecting disadvantaged populations for years. Homelessness, for instance, makes individuals more vulnerable to infection due to crowded living spaces, lack of access to hygienic supplies, and/or healthcare [28]. A study conducted in Boston looked at adults living in a single homeless shelter and found that 36% tested positive for COVID-19 [29]. According to the Johns Hopkins University and American Community Survey, minorities are also disproportionately affected, as the infection rate in predominantly black counties throughout the United States is three times higher than predominantly white; the death rate is an astounding six times higher [9]. Individuals in disadvantaged neighborhoods end up with poorer qualities of education, leading to low income and poorer housing conditions. This exposes them to less sanitary conditions and overcrowding, putting them at higher risks of infection with COVID-19 and many other infections like it [30].

Although not perfectly understood, household composition plays some role in rates of COVID-19 due to lack of social distancing. This can be particularly harmful in homes with elderly populations more susceptible to severe infections [21]. Individuals that heavily rely on public transport have greater chances of contracting COVID-19 due to greater contact with other individuals [30]. This trend of disproportionate outcomes present within populations of low socioeconomic status (SES) extends beyond the United States. In fact, in a study conducted in Stockholm, Sweden, the rate of COVID-19 infection was found to be up to 4 times greater in certain socioeconomically disadvantaged areas than in the rest of the region [31]. Undoubtedly, many socioeconomic factors contribute to the high social vulnerability index and increased COVID-19 exposure and transmission. Overall, this analysis implies that SVI would be a good predictor of transmission and infection rates and perhaps should be considered to estimate the impacts of future pandemics.

As of 2019, approximately 9% of the national population was uninsured, with Floridians at an alarming 13% [32]. The lack of insurance coverage remains an important indicator in health outcomes as those without it tend to experience inadequate quality of care & ultimately poorer health outcomes [33]. Our analysis revealed a substantial increase in cases and death rates among counties with a greater uninsured population. Counties with a high-uninsured population demonstrated a 20.67% greater case rate than the national averages. Our findings were similar to a New York-based study that discovered neighborhoods with larger uninsured populations displayed higher case rates of COVID-19 [11]. Reasons for this may be that uninsured individuals are less likely to seek medical attention while experiencing COVID-19 symptoms, inadvertently exasperating their symptoms. Due to costs, they are also less likely to follow up with proper medical care, leading to a higher fatality rate [34]. The tendency of uninsured patients to postpone care until they are critically ill presents an issue of less effective treatment &, thus, adverse outcomes [35]. The pandemic has also placed a burden on patients through job losses, resulting in the loss of their insurance, making it even more challenging to seek out care [34]. Interestingly, our results also revealed that mortality rates were highest in low-uninsured counties, at 21.85% higher than the FL average and 40.93% higher than the national average. Further research should be done to explore this trend, possibly due to those regions consisting of a larger elderly population, who are already at greater risks of severe complications and mortality in response to COVID-19 and who have health insurance coverage [36].

These trends are somewhat surprising since the United States spends a significant amount of money on health care yet has some of the poorest health outcomes compared to other wealthy countries [30]. The lack of insurance coupled with the shortage in medical equipment and various socioeconomic factors have surely contributed to the high number of covid cases and reveals the United States’ lack of preparation against pandemics [30,37]. On the contrary, countries like China have attempted to make health equity a priority, evident by their response to the pandemic. For instance, in Wuhan, China, they worked to ensure hospitals had ample funds to accept patients without any financial concerns. Some insurance or government subsidy variations covered most patients suffering from COVID-19. Special equipment and procedures were also covered for critically ill patients, as deemed necessary. Overall, the policies implemented ultimately improved access and the effect of treatments of COVID-19. Similarly, Thailand’s extensive & almost universal healthcare coverage allowed free testing and care for insured individuals, eliminating any financial burden one may face. The Ministry of Public Health even extended free testing and treatment to uninsured individuals (i.e., migrant workers who illegally entered and did not have insurance) to mitigate the spread of infection [37]. Although the United States has taken steps towards alleviating the burdens placed on Americans, special attention should be paid to the quality of health insurance policies and options for uninsured individuals. Ultimately, a lack of insurance coverage can have detrimental effects, and eliminating the financial burden on individuals may help to decrease the morbidity from COVID-19.

Overall, the increased rates of COVID-19 infections and mortality highlight the importance of this study in its potential role in helping to mitigate further spread. It can aid public health officials in developing and implementing prevention strategies in these specific vulnerable communities against future epidemics within Florida and the US as a whole.

Study limitations

A significant limitation of this study is the lack of statistical analysis since variance and standard deviations could not be calculated. There was no access to the Florida counties’ information in each tertile of the independent variables. Thus individual county case/death rates could not be accurately matched to tertile averages. As there is no statistical analysis, it should be noted that any presented findings should be critically analyzed for validity. Ultimately, the goal is that the presented information may serve as a guide for planning future statistical analyses.

Population density may also be a considerable limitation, as the epidemiology of an infectious disease is examined. The data is adjusted for population density, as the cumulative case and death values are rates per 100,000 inhabitants. However, dense counties may inherently be more susceptible to increased case rates due to the increased relative proximity of their inhabitants. This may inflate case rates in more urbanized counties. Future studies should analyze case/mortality rates with county population density-independent variables.

 Another significant limitation is the county differences in testing and reporting deaths. For instance, certain counties may be limited in the availability of testing for COVID-19, which may produce inaccurate decreased case rates in these counties. An example would be counties with increased travel distances to testing centers or counties with overcrowded testing centers.

## Conclusions

High levels of poverty and social vulnerability appeared to be clear predictors of COVID-19 case and mortality rates in Florida. Counties with more than 17.3% of the population below the federal poverty line had higher case and mortality rates than Florida and national counties on average. A comparable trend was also observed in Florida counties with a high (>6.67) SVI (ATSDR/CDC social vulnerability index), which indicates that factors such as low socioeconomic status, minority status, language barriers, household composition, disabilities, and poor housing and transportation may all contribute to increased COVID-19 incidence and severity. Average household size did not appear as a clear predictor of COVID-19 outcomes in different counties. Interestingly, Florida counties with a low proportion of uninsured individuals had greatly increased mortality rates, which should be further studied, but is hypothesized to be attributed to a considerable geriatric population with insurance who are concurrently at high mortality risk. Future studies should gain sufficient data to perform a statistical analysis of the findings above. Furthermore, it may be essential to study the individual factors of social vulnerability to look for potential predictors of community outcomes in future COVID-19 or coronavirus epidemics/pandemics.
